# Endothelial expression of human amyloid precursor protein leads to amyloid β in the blood and induces cerebral amyloid angiopathy in knock-in mice

**DOI:** 10.1016/j.jbc.2022.101880

**Published:** 2022-03-31

**Authors:** Yuriko Tachida, Saori Miura, Yui Muto, Hiroyuki Takuwa, Naruhiko Sahara, Akihiro Shindo, Yukio Matsuba, Takashi Saito, Naoyuki Taniguchi, Yasushi Kawaguchi, Hidekazu Tomimoto, Takaomi Saido, Shinobu Kitazume

**Affiliations:** 1 Disease Glycomics Team, Glycobiology Research Group, Global Research Cluster, RIKEN, Saitama, Japan; 2 Department of Clinical Laboratory Sciences, School of Health Sciences, Fukushima Medical University School of Medicine, Fukushima, Japan; 3 Division of Molecular Virology, Department of Microbiology and Immunology, The Institute of Medical Science, The University of Tokyo, Tokyo, Japan; 4 Department of Infectious Disease Control, International Research Center for Infectious Diseases, The Institute of Medical Science, The University of Tokyo, Tokyo, Japan; 5 Research Center for Asian Infectious Diseases, The Institute of Medical Science, The University of Tokyo, Tokyo, Japan; 6 Department of Functional Brain Imaging Research, National Institute of Radiological Sciences, Chiba, Japan; 7 Department of Neurology, Graduate School of Medicine, Mie University, Mie, Japan; 8 Laboratory for Proteolytic Neuroscience, RIKEN Brain Science Institute, Saitama, Japan; 9 Department of Neurocognitive Science, Institute of Brain Science, Nagoya City University Graduate School of Medical Sciences, Nagoya, Japan

**Keywords:** Alzheimer’s disease, cerebral amyloid angiopathy, amyloid β, amyloid precursor protein, Aβ, amyloid β, AD, Alzheimer’s disease, APP, amyloid precursor protein, ARIA, amyloid-related imaging abnormality, BSA, bovine serum albumin, CAA, cerebral amyloid angiopathy, EC, endothelial cell, FSB, 1-fluoro-2,5-bis(3-carboxy-4-hydroxystyryl)benzene, hAPP, human APP, KI, knock-in, PECAM, platelet endothelial cell adhesion molecule, qPCR, quantitative PCR, sAPP, soluble amyloid precursor protein, SLE, systemic lupus erythematosus, Tg, transgenic

## Abstract

The deposition of amyloid β (Aβ) in blood vessels of the brain, known as cerebral amyloid angiopathy (CAA), is observed in most patients with Alzheimer’s disease (AD). Compared with the pathology of CAA in humans, the pathology in most mouse models of AD is not as evident, making it difficult to examine the contribution of CAA to the pathogenesis of AD. On the basis of biochemical analyses that showed blood levels of soluble amyloid precursor protein (APP) in rats and mice were markedly lower than those measured in human samples, we hypothesized that endothelial APP expression would be markedly lower in rodents and subsequently generated mice that specifically express human WT APP (APP770) in endothelial cells (ECs). The resulting EC-APP770^+^ mice exhibited increased levels of serum Aβ and soluble APP, indicating that endothelial APP makes a critical contribution to blood Aβ levels. Even though aged EC-APP770^+^ mice did not exhibit Aβ deposition in the cortical blood vessels, crossing these animals with APP knock-in mice (*App*^NL-F/NL-F^) led to an expanded CAA pathology, as evidenced by increased amounts of amyloid accumulated in the cortical blood vessels. These results highlight an overlooked interplay between neuronal and endothelial APP in brain vascular Aβ deposition. We propose that these EC-APP770^+^:*App*^NL-F/NL-F^ mice may be useful to study the basic molecular mechanisms behind the possible breakdown of the blood–brain barrier upon administration of anti-Aβ antibodies.

Alzheimer’s disease (AD) is a progressive neurodegenerative disorder in humans and the leading cause of dementia. The pathology of AD is characterized by the deposition of amyloid β (Aβ) peptide in the brain parenchyma and blood vessels. Aβ is generated by the two-step proteolytic cleavage of amyloid precursor protein (APP), β-site APP cleaving enzyme-1 (1), and γ-secretase (2), the last of which is composed of four transmembrane proteins (presenilin, nicastrin, Pen2, and Aph1). When APP is alternatively cleaved at the α-site within the Aβ sequence, subsequent γ-secretase cleavage cannot produce neurotoxic Aβ peptide. Alternative mRNA splicing generates three major APP isoforms, APP695, APP751, and APP770, each of which exhibits cell-specific expression. Neurons express APP695 (2), whereas brain vascular endothelial cells (ECs) and platelets express APP770 (3, 4, 5, 6), and APP751 shows a relatively ubiquitous expression pattern.

Cerebral amyloid angiopathy (CAA) is defined as the deposition of Aβ on the vascular walls of the meninges and brain, giving rise to intracerebral hemorrhage and cognitive impairment in the elderly (7). However, epidemiological and clinicopathological data alone do not fully describe the pathological association between CAA and AD.

Impaired clearance and/or overproduction of Aβ lead to its accumulation in the brains of patients with AD (8). Most neuronal Aβ is transported across the blood–brain barrier into the blood (9), and as such, the blood Aβ can derive from a mixture of APP sources including from neurons, smooth muscle cells, ECs, and pericytes, all of which can contribute to vascular Aβ deposition (10). Accumulating evidence shows that several anti-Aβ immunotherapies reduce the amyloid burden in brain parenchyma, while augmenting that of vascular Aβ accumulation (11, 12), indicating that Aβ accumulation in the brain parenchyma and vessels is regulated in different ways. A major therapeutic concern with some anti-Aβ antibodies is the possible breakdown of the blood–brain barrier associated with brain edema and cerebral microhemorrhages, which are observed under magnetic resonance imaging as amyloid-related imaging abnormalities (ARIAs) (13). It is thus important to clarify how brain Aβ accumulates in the vascular walls. Unfortunately, most AD model mice with parenchymal Aβ deposition exhibit only moderate Aβ deposition in the brain blood vessels (14).

Based on our initial study showing that human vascular ECs express a significantly high level of APP770 (3) and that the level of serum soluble amyloid precursor protein (sAPP) in rodents is less than 1% of that in human serum, we explored the possibility that limited endothelial APP expression could result in a markedly reduced CAA pathology in rodent models. In this study, we have developed a novel mouse model that expresses human APP770 (hAPP770) in ECs. We show that the mice exhibited significantly high levels of blood sAPP770 and Aβ40, whereas the accumulation of Aβ in their blood vessels was almost undetectable. However, crossing with APP knock-in (KI) mice resulted in an expanded CAA pathology. These results show that both neuronal and endothelial APP contribute cooperatively to vascular Aβ deposition.

## Results

### Endothelial APP expression in mice

**Figure 1:**
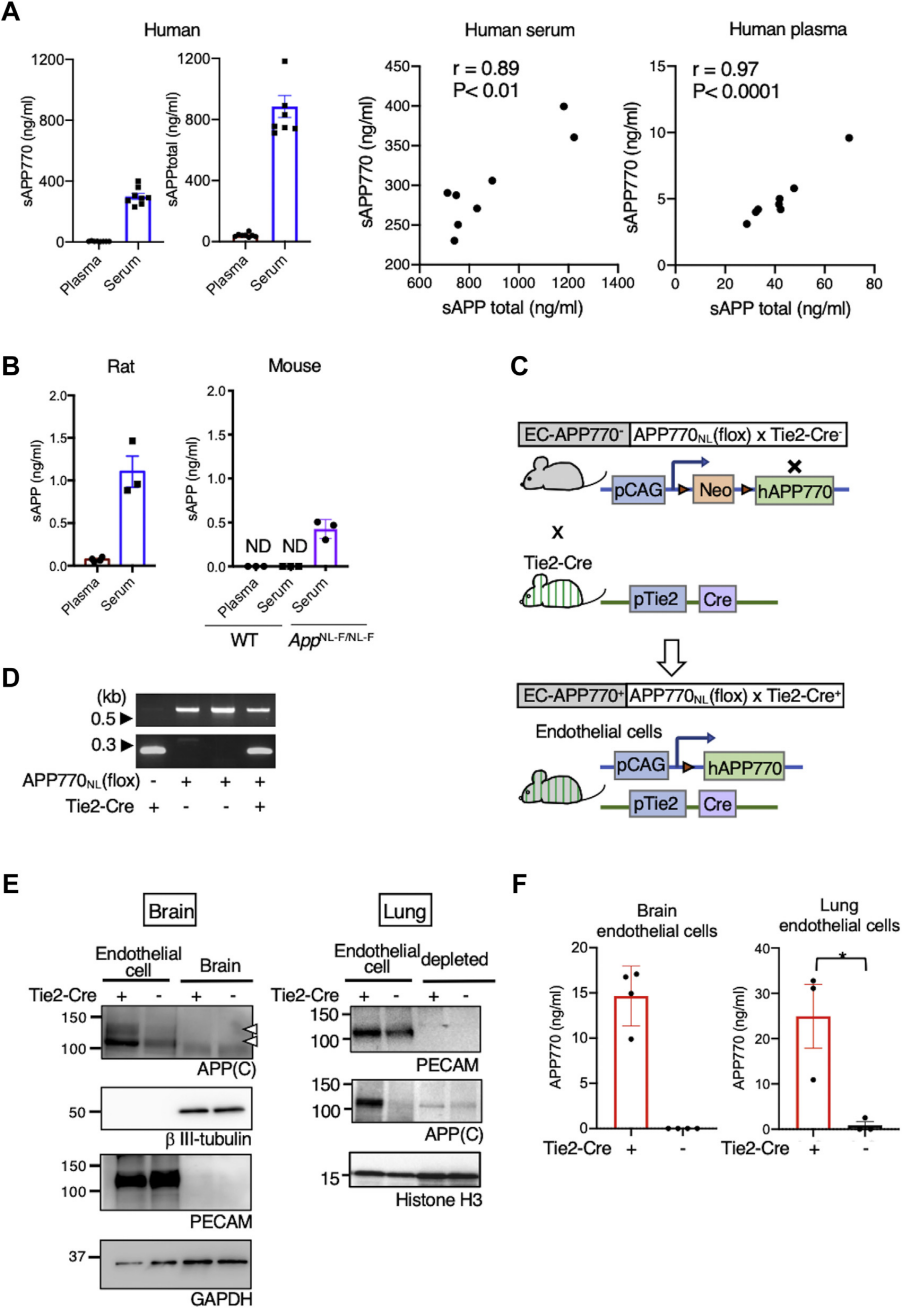
**Endothelial APP expression in mice.***A*, plasma and serum levels of sAPP770 and sAPPtotal in humans (n = 8) were quantified by sandwich ELISA. *Right panels* show scatterplots of sAPPtotal and sAPP770 in human serum and plasma. Using Prism, we calculated Pearson’s correlation coefficient. *B*, biochemical measurement of plasma and serum sAPP levels in 12-week-old rats (n = 3) and WT and *APP*^NL-F/NL-F^ mice (n = 3). Data show mean ± SEM. *C*, schematic drawing showing endothelial-specific hAPP770 expression in mice. *D*, mouse genotype identified by PCR. In the *top image*, the 650 bp band is derived from the APP770-floxed allele. The *bottom image* shows the Tie2-Cre transgene (230 bp band). *E*, endothelial human APP770 expression in EC-APP770^+^ mice. Total brain or lung lysates, or isolated ECs isolated from brains or lungs of 15-month-old EC-APP770^+^ and EC-APP770^−^ mice were analyzed by Western blotting for APP, neuronal marker βIII-tubulin, endothelial marker PECAM, GAPDH, and histone H3. *F*, ECs isolated from brain or lungs from 15-month-old EC-APP770^+^ and EC-APP770^−^ mice (n = 3) were analyzed by human APP770 ELISA. Data show mean ± SEM. APP, amyloid precursor protein; EC, endothelial cell; hAPP, human APP; PECAM, platelet endothelial cell adhesion molecule; sAPP, soluble APP.

We first measured blood sAPP levels in human, rats, and mice. Human serum and plasma were shown to contain 300 and 5.1 ng/ml sAPP770, respectively (Fig. 1*A*). The reason for this difference is that platelets store sAPP770 in α-granules and release it into the serum upon activation (4, 15). We next measured blood sAPPtotal, which accounts for sAPP695, sAPP751, and sAPP770. To our surprise, sAPPtotal in human plasma was also significantly lower (42 ng/ml) than that in serum (890 ng/ml), just like the case of sAPP770, and strong correlations between sAPPtotal and sAPP770 levels in both plasma (*r* = 0.97, *p* < 0.0001) and serum (*r* = 0.89, *p* < 0.01) were found. In contrast, plasma and serum sAPPtotal levels in rats were 70 pg/ml and 1.1 ng/ml, respectively (Fig. 1*B*), which were markedly lower (0.3%) than those measured in human samples. In mice, blood sAPP levels were even undetectable in some cases. Standard AD model mice, *App*^NL-F/NL-F^, showed a slightly higher sAPP770 level in serum, although this still constitutes less than 0.5% of the level in human samples. Even though APP is considered to be expressed ubiquitously (16), we hypothesized that endothelial APP expression might be limited in rodents, which could result in markedly lower levels of blood sAPP and subtle CAA pathology in most AD mouse models. It is considered that a humanized Aβ sequence is necessary for amyloid plaque formation in such models (17). To test our hypothesis, we developed a new mouse model that specifically expresses hAPP770 with the Swedish (KM670/671NL) mutation (18), which facilitates Aβ production, in vascular ECs. We first generated floxed hAPP770_NL_ mice (EC-APP770^−^) under the cytomegalovirus early enhancer/chicken β-actin promoter (Fig. 1, *C* and *D*) and then crossed these mice with *Tie2-Cre* mice, which specifically express Cre recombinase in the ECs by *Tie2* promoter (19), to obtain double-transgenic (Tg) mice (EC-APP770^+^). We used the brains from 15-month-old EC-APP770^+^ and EC-APP770^−^ mice to isolate ECs, which were the cells positive for platelet endothelial cell adhesion molecule (PECAM) and negative for the neuronal marker βIII-tubulin in Western blot analysis (Fig. 1*E*). The results showed that an increased APP signal was not detectable in total brain lysates from either type of mouse, possibly because neurons and glial cells, which are major cell populations in total brains, do not express hAPP770, and ECs are a minor cell population in the brain (20). In contrast, we could detect an increased APP signal in the lysates of brain ECs isolated from EC-APP770^+^ mice. Since an anti-APP antibody, APP(C), detects the carboxy-terminal region of APP that is common to both mouse and hAPP, we quantified the signal intensities of GAPDH and APP in the ECs isolated from EC-APP770^+^ and EC-APP770^−^ mice and estimated the expression level of endothelial hAPP770 in EC-APP770^+^ mice to be about three times higher than that in EC-APP770^−^ mice. We also isolated ECs from the lungs of two types of mice and observed an increased APP signal in EC-APP770^+^ mice. Moreover, ELISA successfully detected hAPP770 in the ECs derived from brains and lungs of EC-APP770^+^ mice (Fig. 1*F*), showing that endothelial hAPP770 expression is not limited to the brain. Taken together, these findings indicate that hAPP770 is specifically expressed in the ECs of EC-APP770^+^ mice.

### EC-APP770^+^ mice exhibit significantly higher level of serum Aβ40/42 but do not exhibit apparent CAA pathology

**Figure 2:**
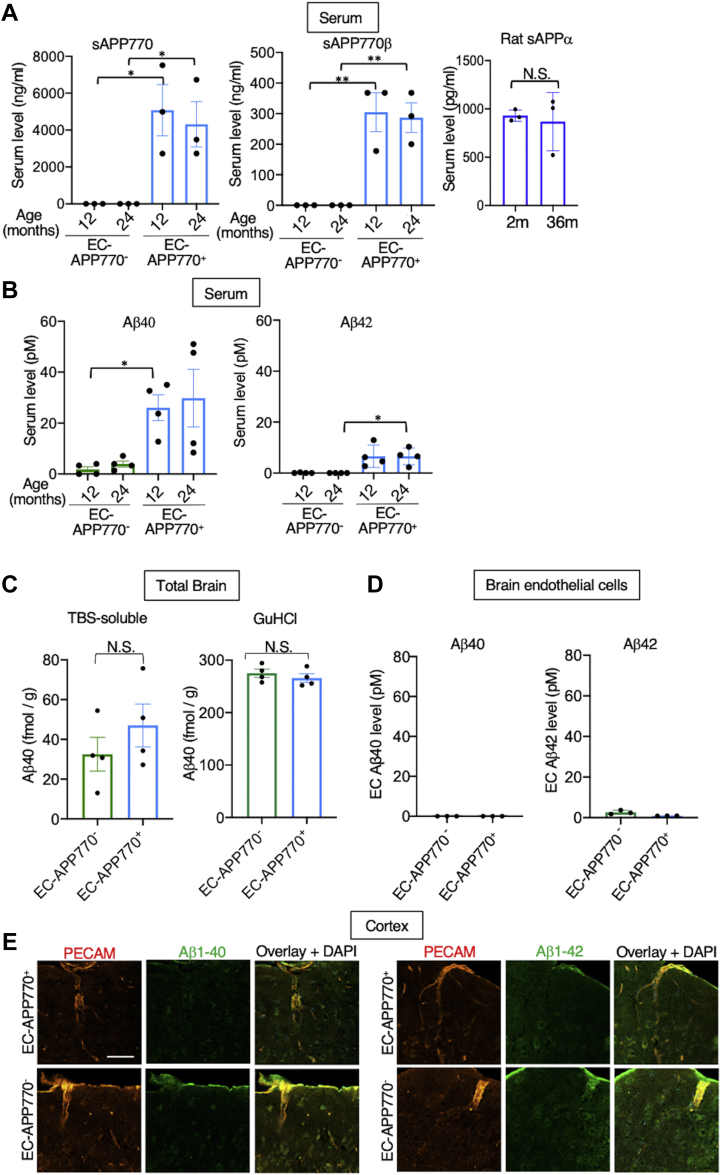
**EC-APP770**^**+**^**mice show increase of serum Aβ40 but do not exhibit apparent CAA pathology.***A*, serum levels of sAPP770 and sAPP770β in 12- and 24-month-old EC-APP770^+^ and EC-APP770^−^ mice (n = 4) are shown as the mean ± SEM. ∗*p* < 0.05, ∗∗*p* < 0.01. One-way ANOVA followed by Tukey’s multiple comparison test. Serum sAPPα in 2- and 36-month-old rats (n = 3) is shown as the mean ± SEM. Not significant (NS) in Student’s *t* test. *B*, serum levels of Aβ40/42 in 12- and 24-month-old EC-APP770^+^ and EC-APP770^−^ mice (n = 4) are shown as the mean ± SEM. ∗*p* < 0.05. One-way ANOVA followed by Tukey’s multiple comparison test. *C*, amount of Aβ40 in the brains of TBS-soluble or GuHCl-extractable fraction from 12-month-old EC-APP770^−^ or EC-APP770^+^ brains (n = 4). NS in Student’s *t* test. *D*, ECs isolated from brains of 15-month-old EC-APP770^+^ and EC-APP770^−^ mice (n = 3) were analyzed by Aβ40/42 ELISA. Data show mean ± SEM. *E*, brain cortex sections from 15-month-old EC-APP770^+^ and EC-APP770^−^ mice were stained for Aβ40/42 with PECAM. The scale bar represents 100 μm. Aβ, amyloid β; APP, amyloid precursor protein; CAA, cerebral amyloid angiopathy; EC, endothelial cell; PECAM, platelet endothelial cell adhesion molecule; sAPP, soluble amyloid precursor protein; TBS, Tris-buffered saline.

A considerable portion of the extracellular region of endothelial APP770 is cleaved for shedding from cultured ECs, and the cells secrete both Aβ40 and Aβ42 in the culture medium (3). We thus anticipated that human sAPP770 and Aβ would be detectable in the serum of EC-APP770^+^ mice. Indeed, serum human sAPP770 levels in 12- and 24-month-old EC-APP770^+^ mice were 4 to 5 μg/ml, whereas they were undetectable in EC-APP770^−^ mice (Fig. 2*A*). We used ELISA, specific to sAPPβ-sw, and measured sAPP770β levels in EC-APP770^+^ mice to be ∼300 ng/ml, whereas sAPP770β was not detectable in EC-APP770^−^ mice. The serum sAPP770β level in EC-APP770^+^ mice is less than 8% of total sAPP770, which compares favorably with the case in humans, in which the sAPPα level is about 15-fold the sAPPβ level (21). No age-dependent difference was detectable for serum sAPP770 or sAPP770β, and we confirmed unaltered serum sAPPα levels in 2- and 36-month-old rats. We also found that serum Aβ40 and Aβ42 levels were markedly higher in EC-APP770^+^ mice than in EC-APP770^−^ mice (Fig. 2*B*). To our knowledge, these data clearly show for the first time that endothelial APP contributes to blood Aβ levels. We did not observe an age-dependent difference in serum Aβ40 and Aβ42 levels between 12- and 24-month-old mice. We next sought to determine whether EC-APP770^+^ mice show increased CAA pathology with aging. We found that steady-state levels of brain Aβ40 were similar between EC-APP770^−^ and EC-APP770^+^ mice (Fig. 2*C*). We then isolated ECs from the brains of EC-APP770^+^ and EC-APP770^−^ mice, but the accumulation of Aβ40/42 in EC-APP770^+^ mice was hardly detectable (Fig. 2*D*). Furthermore, immunohistochemical analyses failed to detect apparent Aβ40/42 signals in cells positive for the endothelial marker PECAM in the brains of 15-month-old EC-APP770^+^ mice (Fig. 2*E*). These results indicate that endothelial APP expression itself does not lead to Aβ deposition in brain blood vessels.

### Exacerbation of CAA pathology by crossing with APP-KI mice

**Figure 3:**
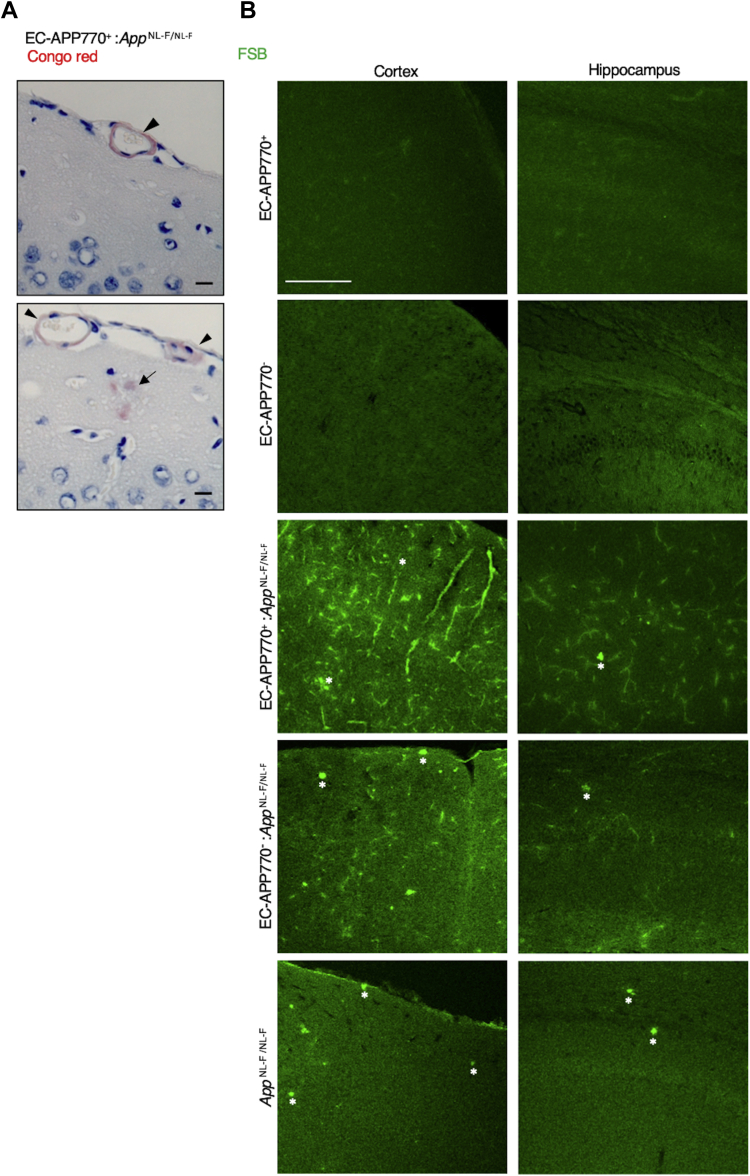

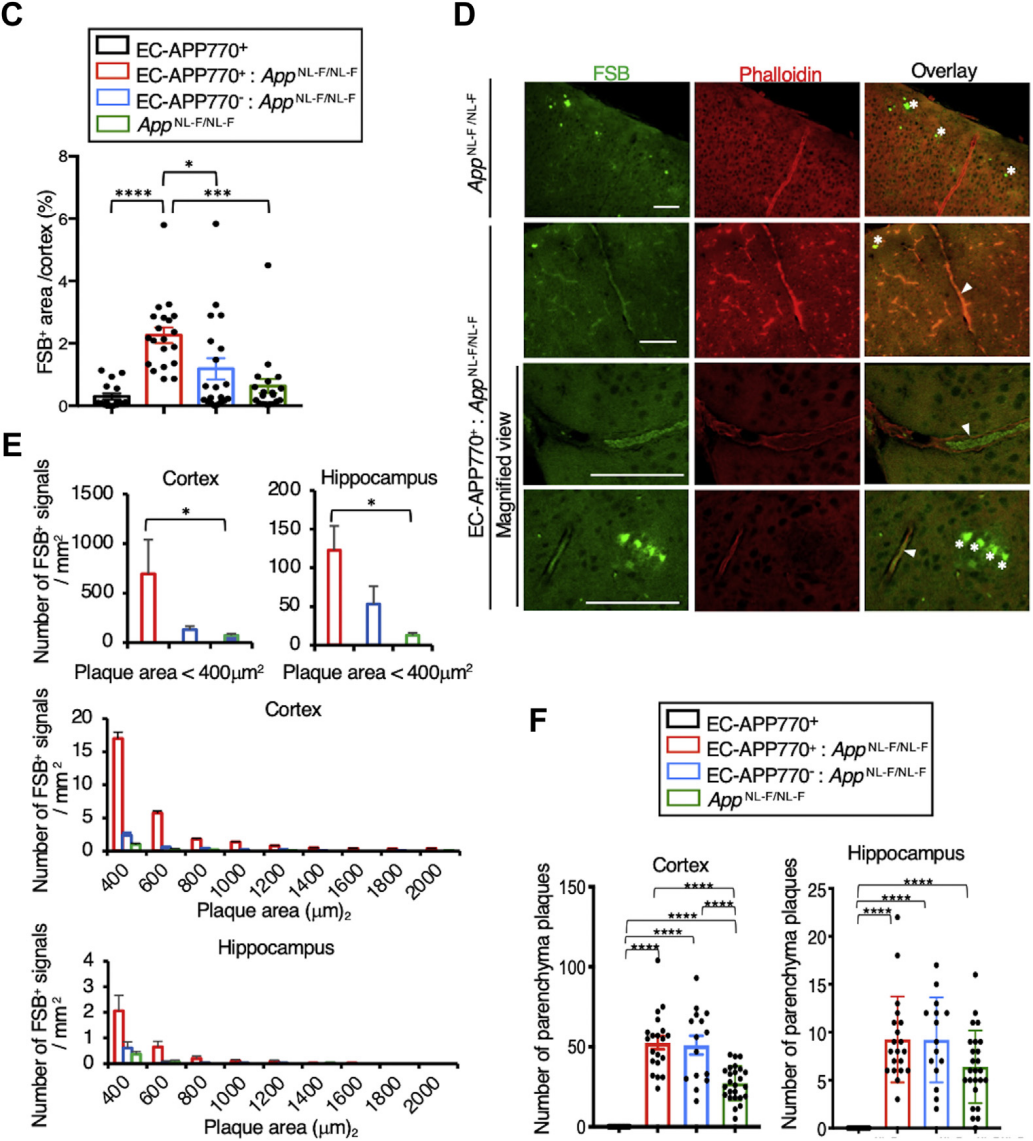
**Offspring of EC-APP770**^**+**^**mice crossed with APP-KI mice exhibit marked Aβ deposition in brain blood vessels.***A*, congo red staining of 4 μm paraffin-embedded sections of 15-month-old EC-APP770^+^:*APP*^NL-F/NL-F^ mouse brains is shown. The scale bar represents 100 μm. *B*, cortical and hippocampal frozen sections from 15-month-old EC-APP770^+^, EC-APP770^−^, EC-APP770^+^:*APP*^NL-F/NL-F^, EC-APP770^−^:*APP*^NL-F/NL-F^, and *APP*^NL-F/NL-F^ mice were stained with FSB. *Asterisks* indicate bright FSB signals, corresponding to typical parenchymal plaques. The scale bar represents 200 μm. *C*, percentage of FSB-positive plaque area per brain cortex is shown as the mean ± SEM. (n = 4). ∗*p* < 0.05, ∗∗∗ *p* < 0.001, ∗∗∗∗*p* < 0.0001, one-way ANOVA with Tukey’s multiple comparison test. *D*, cortical sections from 15-month-old *APP*^NL-F/NL-F^ and EC-APP770^+^:*APP*^NL-F/NL-F^ mice were stained with FSB and Alexa 546-phalloidin. The scale bar represents 100 μm. *Asterisks* and *arrowhead* indicate typical parenchymal plaques and vascular amyloids, respectively. *E*, FSB-positive amyloid plaques in cortical and hippocampal sections from 15-month-old EC-APP770^+^:*APP*^NL-F/NL-F^, EC-APP770^−^:*APP*^NL-F/NL-F^, and *APP*^NL-F/NL-F^ mice were quantified and classified by size and are shown in histogram form. ∗*p* < 0.05, Kruskal–Wallis test with Dunn’s test. *F*, the number of bright FSB signals, parenchymal plaques in 15-month-old EC-APP770^+^, EC-APP770^+^:*APP*^NL-F/NL-F^, EC-APP770^−^:*APP*^NL-F/NL-F^, and *APP*^NL-F/NL-F^ mouse brain hemisphere sections were quantified and are shown as the mean ± SEM (n = 4 mice; five sections were counted per mouse). ∗∗∗∗*p* < 0.00001. One-way ANOVA followed by Tukey’s test. Aβ, amyloid β; APP, amyloid precursor protein; EC, endothelial cell; FSB, 1-fluoro-2,5-bis(3-carboxy-4-hydroxystyryl)benzene; KI, knock-in.

We next considered whether endothelial APP expression influences AD and/or CAA pathology and crossed EC-APP770^+^ mice with AD model mice, *App*^NL-F/NL-F^. Congo red staining of the brain sections of 15-month-old EC-APP770^+^:*App*^NL-F/NL-F^ mice demonstrated the amyloid deposits in the cerebral leptomeningeal vessels, a pathological hallmark of CAA (22), in addition to parenchymal amyloid plaques (Fig. 3*A*). To examine plaque formation in detail, we next used a Congo red derivative, 1-fluoro-2,5-bis(3-carboxy-4-hydroxystyryl)benzene (FSB), on 30 μm frozen sections of 15-month-old EC-APP770^+^, EC-APP770^−^, EC-APP770^+^:*App*^NL-F/NL-F^, EC-APP770^−^:*App*^NL-F/NL-F^, and *App*^NL-F/NL-F^ mouse brains (23). Both EC-APP770^+^ and EC-APP770^−^ mouse brains exhibit negligible FSB signals, whereas *App*^NL-F/NL-F^ mouse brains exhibit FSB-positive parenchymal plaques in both cortex and hippocampus, as previously reported (Fig. 3*B*) (17). A striking feature was observed in EC-APP770^+^:*App*^NL-F/NL-F^ mouse brain, namely, massive amyloid plaque deposition in both cortical and hippocampal regions. Quantitative analysis showed that the percentage of FSB-positive area in the cortex was highest in EC-APP770^+^:*App*^NL-F/NL-F^ mice (Fig. 3*C*). In *App*^NL-F/NL-F^ mice, none of the FSB-positive plaques was colocalized with the signal for phalloidin-Alexa 546, a vascular smooth muscle cell marker, whereas most FSB signals were phalloidin positive in EC-APP770^+^:*App*^NL-F/NL-F^ mice (Fig. 3*D*), indicating that crossing EC-APP770^+^ mice with APP-KI mice results in the robust deposition of amyloid plaques in brain blood vessels. Magnified images of blood vessels showed that FSB signals were present in the luminal regions of phalloidin-Alexa 546-positive vessels. To characterize the amyloid plaques in greater detail, we performed integrated morphometric analysis in which FSB-positive amyloid plaques were counted and classified on the basis of size. Larger amyloid deposits, corresponding to plaque sizes of over 400 μm^2^, were prominent in EC-APP770^+^:*App*^NL-F/NL-F^ mice but not in *App*^NL-F/NL-F^ mice (Fig. 3*D*). This suggested that most parenchymal plaques were less than 400 μm^2^, and large FSB signals were mostly vascular amyloids.

**Figure 4:**
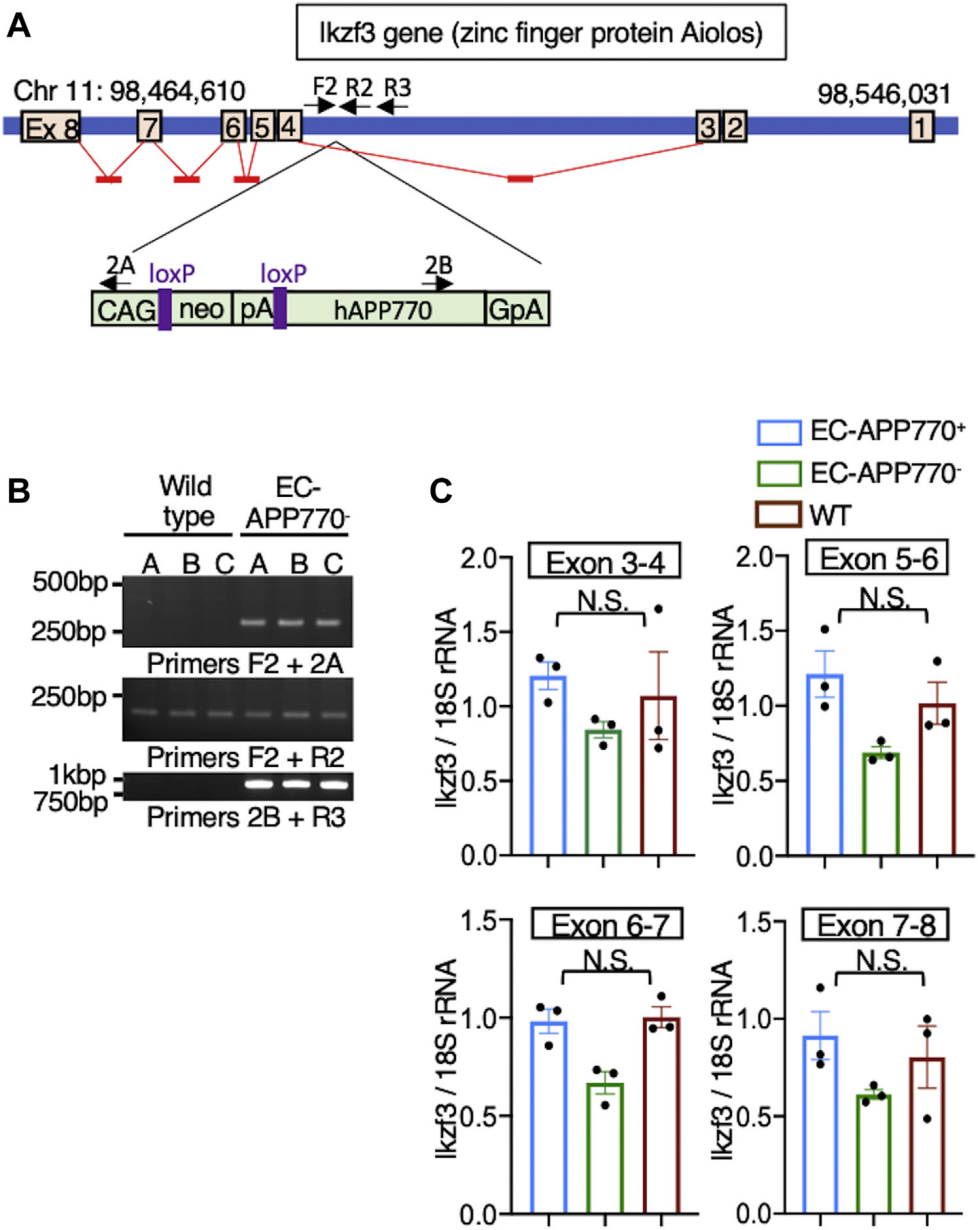
**Identification of integration locus of hAPP770 transgene.***A*, schematic diagram showing hAPP770 transgene located in the Ikzf3 locus on chromosome 11. The positions of primers to validate the insertion site are also shown. *B*, the insertion site was verified by PCR for left junction, inserted genome region, and right junction. *C*, spleens isolated from EC-APP770^+^, EC-APP770^−^, and WT mice were used to quantify Ikzf3 mRNA level by quantitative PCR. Expression levels of Ikzf3 were normalized to corresponding rRNA levels and are shown as the mean ± SEM (n = 3). NS, not significant, one-way ANOVA with Kruskal–Wallis test. EC, endothelial cell; hAPP, human amyloid precursor protein.

In addition to information on their size, parenchymal plaques gave off a bright FSB signal (Fig. 3*D*), and we were able to count these plaques in EC-APP770^+^, EC-APP770^+^:*App*^NL-F/NL-F^, EC-APP770^−^:*App*^NL-F/NL-F^, and *App*^NL-F/NL-F^ mice. The number of parenchymal plaques in cortical regions of EC-APP770^+^:*App*^NL-F/NL-F^ mice was about two times higher than that in *App*^NL-F/NL-F^ mice (Fig. 3*E*). However, as a similar increase was also observed in EC-APP770^−^:*App*^NL-F/NL-F^ mice, we speculated that this could be due to an undesired effect arising from insertion of the floxed hAPP770 transgene. We then decided to determine the integration site of the transgene by next-generation sequencing and found that it was within the intron between exons 3 and 4 of the *Ikzf3* gene in chromosome 11 (Fig. 4*A*). The integration site was confirmed by genomic PCR using primers against genomic DNA and the transgene (Fig. 4*B*). The *Ikzf3* gene encodes Aiolos, a member of the Ikaros family of zinc-finger proteins, which shows restricted expression in the lymphoid system, and regulates lymphocyte differentiation (24). Aiolos expression was not detectable in the brains, and we then measured *Ikzf3* mRNA levels in the spleens of EC-APP770^+^, EC-APP770^−^, and WT mice. Because reference sequence data suggest the presence of Ikzf3 transcript variants, quantitative PCR (qPCR) analysis was performed using multiple primers and probes for different exonic regions of *Ikzf3* mRNA. The mRNA level of splenic Ikzf3 in EC-APP770^−^ mice, determined by a series of primers and probe sets, showed a tendency to be lower than those in WT and EC-APP770^+^ mice, whereas WT and EC-APP770^+^ mice showed similar Ikzf3 mRNA levels (Fig. 4*C*).

### Deposition of Aβ40 in brain blood vessels in aged EC-APP770^+^:*APP*^NL-F/NL-F^ mice

**Figure 5:**
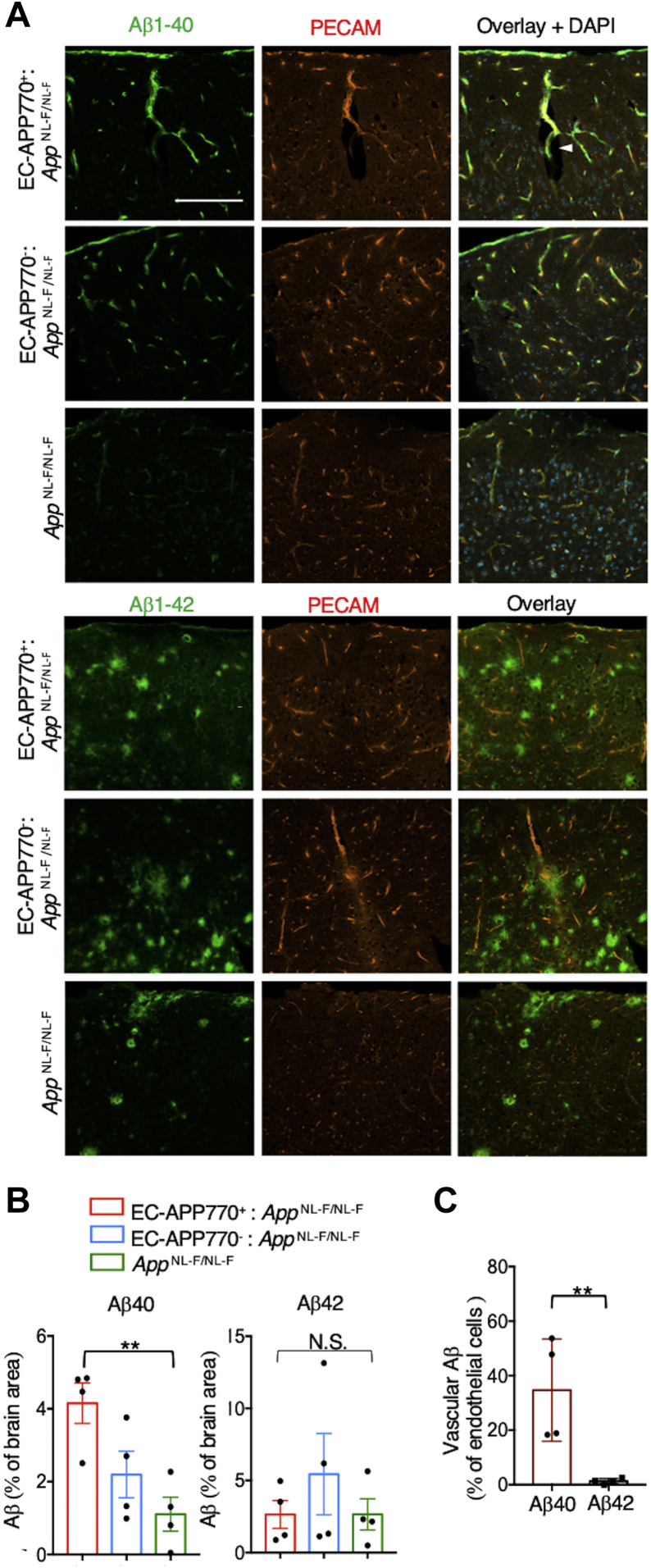
**Deposition of Aβ40 in brain blood vessels in aged EC-APP770**^**+**^**:*APP***^**NL-F/NL-F**^**mice.***A*, brain cortical sections from 15-month-old EC-APP770^+^:*APP*^NL-F/NL-F^, EC-APP770^−^:*APP*^NL-F/NL-F^, and *APP*^NL-F/NL-F^ mice were stained for Aβ40 and Aβ42 with PECAM. An enlarged perivascular space is shown by an *arrowhead*. The scale bar represents 200 μm. *B*, percentage of Aβ40-positive and Aβ42-positive areas per brain cortex is shown as the mean ± SEM. (n = 4). ∗∗*p* < 0.01, NS, not significant, in one-way ANOVA with Tukey’s multiple comparison test. *C*, from the analysis of immunostained images of EC-APP770^+^:*APP*^NL-F/NL-F^ brain sections, percentage of PECAM^+^Aβ^+^ signals relative to total PECAM^+^ signals (vascular Aβ) is shown as the mean ± SEM (n = 4). ∗∗*p* < 0.01, Student’s *t* test. Aβ, amyloid β; APP, amyloid precursor protein; EC, endothelial cell; PECAM, platelet endothelial cell adhesion molecule.

As biochemical analyses previously showed that Aβ isoforms shorter than 42 residues form a component of the cerebrovascular amyloid from patients with AD (25), we next performed immunohistochemical analyses on brain sections from EC-APP770^+^:*App*^NL-F/NL-F^, EC-APP770^−^:*App*^NL-F/NL-F^, and *App*^NL-F/NL-F^ mice to detect different Aβ species. Strong Aβ42 and weak Aβ40 signals were found in *App*^NL-F/NL-F^ mice (Fig. 5*A*), as has been reported previously (17). Meanwhile, both EC-APP770^+^:*App*^NL-F/NL-F^ and EC-APP770^−^:*App*^NL-F/NL-F^ mice exhibited large numbers of Aβ40 and Aβ42 plaques. Quantitative analysis showed that significantly higher levels of Aβ40 were accumulated in EC-APP770^+^:*App*^NL-F/NL-F^ mice than in *App*^NL-F/NL-F^ mice, whereas the accumulation of Aβ42 did not differ in these mice (Fig. 5*B*). Notably, most of the signal of Aβ40, but not of Aβ42, was colocalized with that of PECAM, indicating that the deposition of Aβ40 is restricted to brain blood vessels, whereas Aβ42 signal was observed exclusively in the brain parenchyma; quantitative analysis supported this observation (Fig. 5*C*). Notably, enlarged perivascular spaces, suggestive CAA indicators (26), are observed in the brains of EC-APP770^+^:*App*^NL-F/NL-F^ (Figs. 5*A* and S1). These results also suggest that endothelial APP expression in AD model mice exacerbates CAA pathology.

## Discussion

Particular Aβ isoforms in blood have recently been reported to be valuable biomarkers for AD diagnosis (27). In our study, endothelial hAPP expression in mice led to a dramatic increase of serum Aβ levels, suggesting that blood Aβ is mostly derived from endothelial APP770. Given that the levels of cerebrospinal fluid Aβ, plasma Aβ, and brain Aβ are highly correlated in patients with AD, endothelial Aβ production could be related to not only endothelial dysfunction but also AD progression (28, 29).

It remains unclear whether the Aβ in accumulated plaque in cortical blood vessels is derived from the circulation, the blood vessel wall itself, or the central nervous system (30). High levels of plasma Aβ do not cause CAA in Tg mice expressing signal peptide plus the carboxyl-terminal region of APP that contains Aβ (31). Nevertheless, as intraperitoneal inoculation with Aβ-rich brain extracts (possibly containing Aβ oligomers) from AD model mice can induce CAA in APP-overexpressing mice (32), circulating Aβ oligomers may play a seeding-related or transmission-related role in vascular Aβ deposition. Neuronal APP-expressing mice also exhibit a mild CAA pathology, supporting the notion that neuronal Aβ could be a source of CAA. In this study, we show for the first time that the endothelial expression of hAPP without Dutch/Iowa mutation contributes to vascular Aβ deposition in mice. By simply expressing hAPP in ECs, the resulting EC-APP770^+^ mice did not exhibit an apparent CAA pathology. Remarkably, however, by crossing the mice with hAPP KI mice, in which neuronal APP was expressed, the CAA pathology was markedly exacerbated. These results indicate that both endothelial APP and neuronal APP cooperatively contribute to the progression of CAA pathology. One of the possible explanations for this is that an undetectable level of vascular Aβ deposits derived from endothelial APP becomes seeds for accelerating Aβ accumulation in cerebral blood vessels. Interestingly, Xu *et al.* (33) showed that crossing classical AD model mice, Tg2576, with Tg-SwDI mice, which express Dutch-E22Q and Iowa-D23N APP mutant by Thy1.2 promoter and exhibit low levels of vascular Aβ deposits, resulted in the massive deposition of vascular amyloid (34). In aged EC-APP770^+^:*App*^NL-F/NL-F^ mice, deposition of Aβ in the walls of cortical arteries is prominent, and such vascular Aβ deposition could impair the normal perivascular drainage of Aβ from the brain parenchyma (35). Even though Aβ42 has a higher propensity than Aβ40 to aggregate, immunohistochemical analyses of EC-APP770^+^:*App*^NL-F/NL-F^ mice showed that the vascular amyloid consisted mostly of Aβ40, as has been reported in the case of patients with AD (25), whereas parenchymal amyloid consisted mostly of Aβ42. It is conceivable that Aβ40-rich vascular amyloids possess a relatively immature β-pleated structure and weakly react with FSB. The finding supports the idea that highly aggregative nature of Aβ42 hardly enter the perivascular drainage pathway but fibrillizes in plaques, and Aβ40 accumulated in the walls of blood vessels in the course of its perivascular drainage (36). Another interesting possibility is that ECs in the aged mice could induce specific molecule(s) with high affinity for the Aβ40 oligomer. Furthermore, decreased levels of cerebrospinal fluid and blood Aβ40/42 have been reported in patients with AD. Even though vascular amyloids in EC-APP770^+^:*App*^NL-F/NL-F^ mice are detected with anti-Aβ40 but not with anti-Aβ42 antibody, it is still conceivable that a low level of Aβ42 could be recruited to vascular amyloids, leading to a reduction of blood Aβ42.

Several kinds of anti-Aβ antibodies have been developed as AD immunotherapeutics (37), of which aducanumab has now been approved by the US Food and Drug Administration to treat AD. However, the administration of these anti-Aβ antibodies often causes ARIAs, suggestive of signals for vasogenic edema and sulcal effusions (ARIA-E) and microhemorrhage and hemosiderin deposits (ARIA-H), as adverse effects. The EC-APP770^+^:*App*^NL-F/NL-F^ mice would be useful to study the degree of ARIA induced by the administration of anti-Aβ antibodies and the basic molecular mechanism of ARIA. A major advantage of EC-APP770^+^ mice is that the efficacy of anti-Aβ therapeutics can be easily determined by monitoring blood Aβ levels.

An insertion of the floxed APP transgene within the intron of the gene encoding Aiolos, *Ikzf3*, results in a tendency for decreased splenic Aiolos expression in EC-APP770^−^ mice. Notably, however, such tendency is somewhat canceled in EC-APP770^+^ mice, possibly because of a shorter length of the transgene. Aiolos overexpression has been reported in B cells from patients with systemic lupus erythematosus (SLE) (38), whereas it was recently shown that the cerebral small blood vessel disease burden is increased in SLE (39). By crossing with AD model mice, not only EC-APP770^+^ but also EC-APP770^−^ mice exhibited amyloid plaques in the cerebral small blood vessels, and such abnormality observed in EC-APP770^−^ mice is likely related to the SLE-like features. Further to this, the endothelial-specific angiopoietin 2–Tie2 ligand–receptor system has been identified as a major regulator of endothelial inflammation in SLE. Mating female EC-APP770^−^ mice with WT male mice should be avoided, since this results in offspring that express APP770 throughout the body, possibly because of aberrant Tie2 activation in the pregnant mice (40).

Taking these findings together, our newly developed EC-APP770^+^ mice, combined with AD model mice, *App*^NL-F/NL-F^, could serve as a useful research tool to study the disease mechanism underlying CAA pathology and the mechanistic interactions between CAA and AD.

## Experimental procedures

### Materials

The sources of materials used in this study were as follows: tissue culture medium and reagents, including Dulbecco’s modified Eagle’s medium, were from Invitrogen; all other chemicals were from Sigma or Wako Chemicals. Detailed information on the primary and secondary antibodies used in this study is provided in Table S1. pCALNL5 (RDB01862; RIKEN BioResource Center) plasmid was from Dr I. Saito (University of Tokyo).

### Animal use and care

All animal experiments were approved by the Institutional Animal Care and Use Committee at RIKEN, Fukushima Medical University, or the National Institute of Radiological Sciences in compliance with their respective guidelines for animal experiments.

### Generation of EC-APP770^+^ mice

Endothelial APP770-overexpressing mice (EC-APP770^+^) were generated with the help of the Support Unit for Animal Resources Development in RIKEN BSI. The plasmid pCANLN5-hAPP770 was generated by inserting the hAPP770 Swedish mutant (hAPP770_NL_) into the pCALNL5 vector (41). The resulting plasmid has the neomycin-resistant (neoR) gene flanked by two loxP sites under the cytomegalovirus early enhancer/chicken β-actin promoter (CAGp), followed by the hAPP770Sw sequence. pCANLN5-hAPP770 was linearized and injected into C57BL/6J pronuclear zygotes. We extracted DNA from ear punches of mouse pups and identified nine F_1_ generation mice by PCR using the following cocktail of primers (17): 5′-ATCTCGGAAGTGAAGATG-3′, 5′-ATCTCGGAAGTGAATCTA-3′, 5′-TGTAGATGAGAACTTAAC-3′, and 5′-CGTATAATGTATGCTATACGAAG-3′. Heterozygous EC-APP770^−^ mice were then crossed with Tie2-Cre mice (19). The *APP770*_*NL*_ allele was identified by PCR (forward, 5′-AGCCGCAGCCATTGCCTTTTATGGTA-3′, reverse, 5′-CAGGTCGGTCTTGACAAAAAGAACCG-3′ for *APP770*_*NL*_). The *Tie2-Cre* allele was identified by PCR (forward, 5′-ACATGTTCAGGGATCGCCAG-3′, reverse, 5′-TAACCAGTGAAACAGCATTGC-3′). To obtain EC-APP770^+^ and EC-APP770^−^ mice, male EC-APP770^+^ mice were mated with WT C57BL/6 female mice. EC-APP770^+^ mice were crossed with AD model (*App*^NL-F/NL-F^) mice (17).

### Determination of the integration site of the APP transgene

The location of insertion of the hAPP770 transgene was determined using Illumina NGS (Hokkaido System Science Co, Ltd). The source code to perform the integration site analysis (42) is available at https://github.com/hbc/li_hiv. Genomic PCR analyses were performed to confirm the insertion loci using F2 (5′-CTTCTGTTGTTTGGCTTTAT-3′), 2A (5′-GACGTCAATGGAAAGTCCCT-3′), R2 (5′-GTTGGTCTCATGAGGCAAGT-3′), 2B (5′-GACGTCAATGGAAAGTCCCT-3′), and R3 primers (5′-TGTGTGTGGCAAACACTTTG-3′).

### Immunohistochemical and histochemical studies

To prepare brain sections, mice were anesthetized with medetomidine–midazolam–butorphanol and then transcardially perfused with PBS first and then with 0.1 M phosphate-buffered 4% paraformaldehyde. Brains were removed and sequentially immersed in the same fixative for 16 h. Congo red staining was performed on 4 μm-thick paraffin-embedded brain sections. To prepare frozen sections, fixed brains were immersed in phosphate-buffered 30% sucrose for 16 h at 4 °C. Sagittal or coronal (thickness of 30 μm) sections were immunostained using the floating method. Briefly, sections were permeabilized with 0.3% Triton X-100 in 1% bovine serum albumin (BSA)–PBS for 30 min first and then with 5% goat serum–PBS for 60 min. The sections were then incubated with primary and secondary antibodies and 4′,6-diamidino-2-phenylindole. Detailed descriptions of the primary and secondary antibodies used are provided in Table S1. For the detection of amyloidosis, FSB was used (23). The sections were then blocked with 1% BSA–PBS containing 0.1% Tween-20 for 30 min, followed by incubation with phalloidin-Alexa 546 (Invitrogen) in 1% BSA–PBS for 30 min (43). The stained sections were observed using an Olympus FV-1000 confocal microscope. We quantified immunoreactive areas using MetaMorph imaging software (Universal Imaging Corp), as previously described (17). To reduce the variance among tissue sections, data were averaged from at least four sections per mouse.

### Isolation of ECs

After perfusion with ice-cold PBS, brains/lungs were dissociated using Adult Brain/Lung Dissociation kit (Miltenyi Biotech) and gentleMACS Dissociator with Heater (Miltenyi Biotech), in accordance with the established protocol. ECs were then isolated with CD31 MicroBeads (Miltenyi Biotech) using AutoMACS (Miltenyi Biotech) or using anti-CD146 antibody (BioLegend; clone: ME-9F1) coupled with Dynabeads Sheep anti-Rat IgG (Thermo Fisher Scientific) (3).

### Western blotting

Cells from mouse brain or spleen tissues were lysed with T-PER Tissue Protein Extraction Reagent (Thermo) containing Complete Protease Inhibitor Cocktail (Roche Applied Science). Protein concentrations were determined with bicinchoninic acid protein assay reagents (Pierce). Samples (25–40 μg of protein) were subjected to SDS-PAGE (5–20% gradient gel) and transferred to nitrocellulose membranes. For Western blot analyses, following incubation with 5% nonfat dried milk in Tris-buffered saline–containing 0.1% Tween-20, the membranes were incubated with primary antibodies and horseradish peroxidase–labeled secondary antibodies. Signals were detected with SuperSignal West Dura Extended Duration Substrate (Thermo Fisher Scientific) using an ImageQuant LAS-4000 Mini imager (GE Healthcare). Intensities of the resultant protein bands were quantified using ImageQuant TL software (GE Healthcare).

### Quantification of sAPP and Aβ

To measure sAPPtotal, sAPP770, sAPP770β, Aβ40, and Aβ42 in plasma or serum, a Human sAPP Total Assay Kit, a Human APP770 Assay Kit, an sAPPβ-sw Assay Kit (highly sensitive) (IBL-Japan), a Human/Rat β-amyloid (40) ELISA kit, and a Human/Rat β-amyloid (42) assay kit High-Sensitive (Wako) were used, respectively. To measure rat sAPP, a Mouse/Rat sAPPα (highly sensitive) Assay Kit (IBL-Japan) was used. A mouse complement component C5a DuoSet ELISA (catalog no.: DY2150; R&D Systems) was used to measure serum C5a in mice.

### RNA preparation

For real-time PCR, spleens isolated from mice were used to isolate total RNA using TriPure Isolation Reagent (Roche). RNA samples (1–5 μg) were reverse transcribed with random hexamers using a Transcriptor First-Strand cDNA Synthesis kit (Roche), in accordance with the manufacturer’s protocol.

### Real-time PCR analysis

The amount of complementary DNA of specific genes was quantified using TaKaRa qPCR probe (TaKaRa) or the Universal ProbeLibrary (Roche) with TaqMan Master (Roche) and the LightCycler 96 system (Roche), in accordance with the manufacturer’s instructions. The primer and probe sequences for *Ikzf3* are shown in Table 1, and those for 18S rRNA were 5′-GCAATTATTCCCCATGAACG-3′, 5′-GGGACTTAATCAACGCAAGC-3′, and ProbeLibrary probe 48 (Roche). The probe for *Ikzf3* was labeled with the fluorescent reporter dye fluorescein amidite at its 5ʹ end and the quencher dye Black Hole Quencher-1 at its 3ʹ end. The probe for rRNA was labeled with 2′-chloro-7′phenyl-1,4-dichloro-6-carboxy-fluorescein at its 5ʹ end and the quencher dye Black Hole Quencher-1 at its 3ʹ end. The relative amount of each gene expression was calculated using the comparative cycle threshold (2^−ΔΔCt^) method (44).

**Table 1:** Sequences of qPCR primers and probe for Ikzf3

Targeting region	Primer and probe sequence (5′-3′)
Exon 3–4	F: CAGCTTGCCCAAACCTCATG
	R: GCTGTATTCATCTTTCACTTTCATCG
	FAM-TCTTCTCCTGCATCTTCGTCTTCATTGGCT-BHQ-1
Exon 5–6	F: GTGAGTTCTGCGGAAGAAGCTA
	R: GCTCTCTCACTTCCCATCTCG
	FAM-TGTCTTGCCTCCACACTTGCAGCGTC-BHQ-1
Exon 6–7	F: GGAGGCAAGACACATCAAAGC
	R: CGGGATTGTAGTTGGCATCGA
	FAM-CAGTGCCGCTTCTCACCGATGAATTTCTGA-BHQ-1
Exon 7–8	F: TCTCGTCCTGGACAGATTAGCA
	R: GTAGCCGGGATTGTAGTTGGC
	FAM-CAGTGCCGCTTCTCACCGATGAATTTCTGA-BHQ-1

Abbreviations: BHQ-1, Black Hole Quencher-1; F, forward; FAM, fluorescein amidite; R, reverse.

### Statistical analysis

All data are shown as mean ± SEM. For comparison between two groups, statistical analyses were performed by Student’s *t* test. Multiple comparison tests were performed using GraphPad Prism 7 software (GraphPad Software, Inc). Normality was tested using a D’Agostino-Pearson normality test. For comparisons among three or more groups, one-way ANOVA followed by a post hoc test (Tukey’s multiple comparison test) or Bonferroni’s multiple comparison test was performed. The data were collected and processed in a randomized and blinded manner.

## Data availability

All relevant data are available from the corresponding author upon reasonable request.

## Supporting information

This article contains supporting information.

## Conflict of interest

The authors declare that they have no conflicts of interest with the contents of this article.
