# Supplementary material for: Endothelial expression of human amyloid precursor protein leads to amyloid β in the blood and induces cerebral amyloid angiopathy in knock-in mice
Source: J Biol Chem. 2022 Mar 31;298(6):101880. doi: 10.1016/j.jbc.2022.101880 (PMC9144051; doi:10.1016/j.jbc.2022.101880)
Supplement: Supplemental Table S1 [file mmc1.docx]

**Supplementary Table 1. Antibodies used in this study**

| **Primary antibody** | **Secondary antibody** | **Application** |
| --- | --- | --- |
| Rabbit anti-APP(C) | HRP-conjugated anti-rabbit | Western blot analysis in Figure 1e |
| (IBL-Japan, #18961, 1:100) | (GE Healthcare, NA934, 1:3000) |  |
| Rabbit anti-beta III tubulin | HRP-conjugated anti-rabbit | Western blot analysis in Figure 1e |
| (Abcam, ab18207, 1:400) | (GE Healthcare, NA934, 1:3000) |  |
| Rat anti-mPECAM | HRP-conjugated anti-rat | Western blot analysis in Figure 1e |
| (Biolegend, MEC13.1, 1:1000) | (GE Healthcare, NA935, 1:3000) |  |
| mouse anti-GAPDH | HRP-conjugated anti-mouse | Western blot analysis in Figure 1e |
| (Merck, mAB374, 1:1000) | (GE Healthcare, NA931, 1:3000) |  |
| Rabbit anti-histone H3 | HRP-conjugated anti-rabbit | Western blot analysis in Figure 1e |
| (Abcam, ab1791, 1:3000) | (GE Healthcare, NA934, 1:3000) |  |
| Rat anti-mPECAM | Alexa546-donkey anti-rat | Immunofluorescent detection in Figure 2e, 5a |
| (Biolegend, MEC13.1, 1:50) | (Thermo, A-11030, 1:300) |  |
| Rabbit anti-amyloid β(1-40) (IBL-Japan, #18580, 1:100) | Alexa488-donkey anti-rabbit  (Thermo, A-11008, 1:100) | Immunofluorescent detection in Figure 2e, 5a |
| Rabbit anti-amyloid β(1-42) | Alexa488-donkey anti-rabbit | Immunofluorescent detection in Figure 2e, 5a |
| (IBL-Japan, #18582, 1:100) | (Thermo, A-11008, 1:100) |  |
| Alexa546- Phalloidin  (Thermo, A22283, 1:40) |  | Immunofluorescent detection in Figure 3c |
