# Supplementary material for: Endothelial expression of human amyloid precursor protein leads to amyloid β in the blood and induces cerebral amyloid angiopathy in knock-in mice
Source: J Biol Chem. 2022 Mar 31;298(6):101880. doi: 10.1016/j.jbc.2022.101880 (PMC9144051; doi:10.1016/j.jbc.2022.101880)
Supplement: Supplemental Figure S1 [file mmc2.pdf]

Fig S1

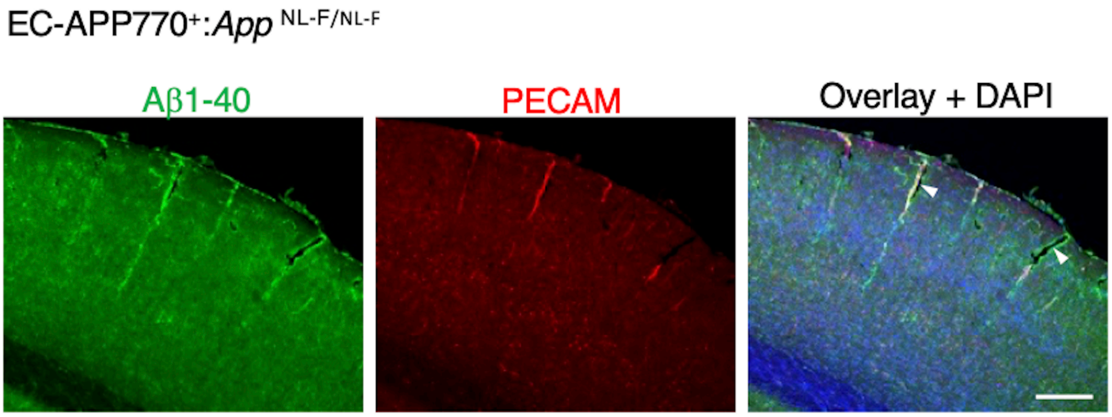

Figure S1 legend
Brain cortical sections from 15-month-old EC-APP770^{+}:*APP*$^{NL-F/NL-F}$ mice were stained for Aβ40 with PECAM. Enlarged perivascular spaces are shown by arrowheads. Scale bar: 200 μm.
